# A Live‐Cell Epigenome Manipulation by Photo‐Stimuli‐Responsive Histone Methyltransferase Inhibitor

**DOI:** 10.1002/advs.202404608

**Published:** 2024-09-09

**Authors:** Chuan‐Shuo Wu, Xin Sun, Li Liu, Liang Cheng

**Affiliations:** ^1^ Beijing National Laboratory for Molecular Sciences (BNLMS) CAS Key Laboratory of Molecular Recognition and Function CAS Research/Education Center for Excellence in Molecular Sciences Institute of Chemistry Chinese Academy of Sciences Beijing 100190 China; ^2^ University of Chinese Academy of Sciences Beijing 100049 China; ^3^ State Key Laboratory of Elemento‐Organic Chemistry Nankai University Tianjin 300071 China

**Keywords:** chemical modulation, histone modifications, lysine methylation, lysine methyltransferases, photo‐responsive

## Abstract

Post‐translational modifications on the histone H3 tail regulate chromatin structure, impact epigenetics, and hence the gene expressions. Current chemical modulation tools, such as unnatural amino acid incorporation, protein splicing, and sortase‐based editing, have allowed for the modification of histones with various PTMs in cellular contexts, but are not applicable for editing native chromatin. The use of small organic molecules to manipulate histone‐modifying enzymes alters endogenous histone PTMs but lacks precise temporal and spatial control. To date, there has been no achievement in modulating histone methylation in living cells with spatiotemporal resolution. In this study, a new method is presented for temporally manipulating histone dimethylation H3K9me2 using a photo‐responsive inhibitor that specifically targets the methyltransferase G9a on demand. The photo‐caged molecule is stable under physiological conditions and cellular environments, but rapidly activated upon exposure to light, releasing the bioactive component that can immediately inhibit the catalytic ability of the G9a in vitro. Besides, this masked compound could also efficiently reactivate the inhibition of methyltransferase activity in living cells, subsequently suppress H3K9me2, a mark that regulates various chromatin functions. Therefore, the chemical system will be a valuable tool for manipulating the epigenome for therapeutic purposes and furthering the understanding of epigenetic mechanisms.

## Graphical abstract

Histone H3 tail modifications regulate chromatin structure and gene expression. Current tools cannot edit native chromatin, and small molecules lack precise control. The study presents a method for temporally manipulating histone H3K9me2 using a photo‐responsive inhibitor targeting G9a methyltransferase. This stable, light‐activated compound precisely controls methylation in living cells, aiding therapeutic and epigenetic research.

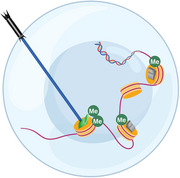

## 1 Introduction

Histones are essential proteins that tightly compact and organize DNA into chromosomes. Modifying histones is a crucial post‐translational process that significantly influences gene expression. Researchers have so far identified several distinct post‐translational modifications (PTMs) within histones. Among these, acetylation, methylation, phosphorylation, and ubiquitylation are the best understood, while GlcNAcylation, citrullination, crotonylation, and isomerization are much newer discoveries that are still undergoing thorough investigations. These modifications alter chromatin structure and recruit histone modifiers, ultimately impacting various cellular processes, including transcription activation/inactivation, chromosome organization, DNA damage, and DNA repair.^[^ ^1^, ^2^, ^3^ ^]^ Additionally, those alterations are also being linked to the diagnosis of neurological conditions such as schizophrenia and Alzheimer's disease, as well as the prognosis of various cancer types.^[^ ^4^, ^5^, ^6^ ^]^

In the last decades, advancements in the manipulation of those epigenetic marks have allowed us to experimentally and directly assess the functional significance of those modifications that accumulate at specific genomic regions. For instance, the application of unnatural amino acid integration,^[^ ^7^, ^8^ ^]^ protein splicing,^[^ ^9^, ^10^ ^]^ fusion proteins,^[^ ^11^, ^12^ ^]^ and sortase‐based editing^[^ ^13^ ^]^ has made it possible to manipulate various histones PTMs in cellular environments, although most of these methods are not suitable for modifying native chromatin. An alternative approach that has garnered increasing attention is the manipulation of PTMs using highly potent and selective small molecules. These compounds can influence specific histone modifications by inhibiting the catalytic activity of the modifying enzymes (writers and erasers), allowing for the assessment of downstream consequences to investigate the involvement and biological functions of those modifications. Consequently, those small organic modulators are considered crucial tools for understanding the roles of epigenetic modification pathways and are essential for validating “druggable” targets in pre‐clinical studies, both in academic and industry settings.^[^ ^14^, ^15^, ^16^ ^]^ For instance, several inhibitors targeting the histone deacetylase enzymes (HDACs) have received approval for cancer treatment, and additional candidates are currently undergoing clinical evaluation.^[^ ^17^, ^18^, ^19^, ^20^, ^21^, ^22^, ^23^, ^24^, ^25^, ^26^, ^27^ ^]^ In the meantime, the discovery and development of small organic inhibitors targeting histone lysine methyltransferases (KMTs) and demethylases (KDMs) have recently emerged as a dynamic and rapidly expanding research field.^[^ ^28^, ^29^, ^30^, ^31^, ^32^, ^33^ ^]^ These molecules have been shown to play a significant role in the regulation of gene expression, which is linked to the development of various human cancers and other diseases.^[^ ^34^, ^35^, ^36^, ^37^, ^38^, ^39^ ^]^

**Scheme 1:**
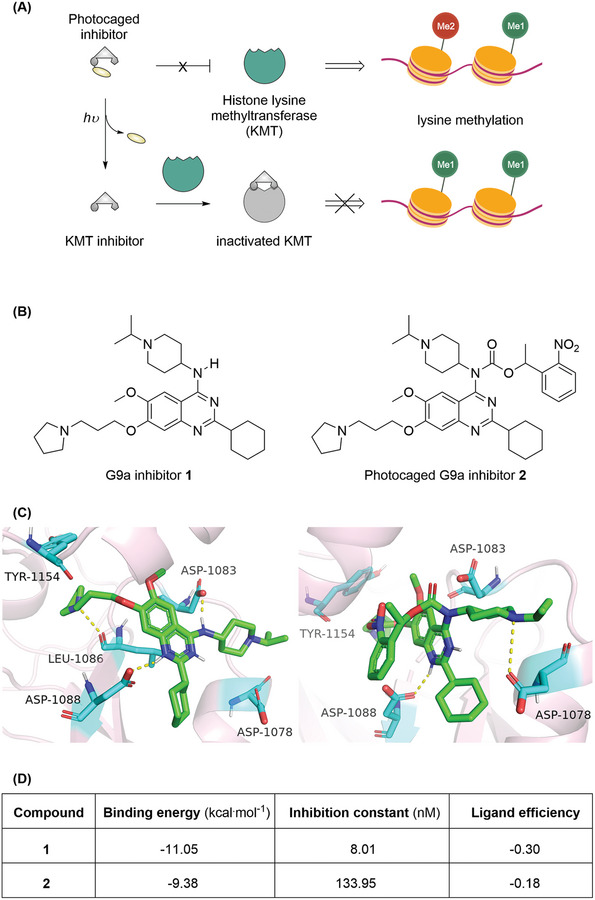
A) Schematics of photo‐induced inhibition of histone lysine methyltransferase (KMT) and hence modulation of the methylation with photo‐activatable small organic inhibitor. B) Chemical structures of G9a inhibitor UNC0638 **1** and its photo‐caged precursor **2**. C) Representative images of the docked structures of compounds **1** (left) and **2** (right) within the G9a (PDB ID: 3RJW) in the corresponding binding pockets. The interactive amino acid residues in the binding pockets and the related hydrogen binding were presented in dot lines. D) Molecular docking analysis of compounds **1**–**2** within the binding sites of G9a.

In recent years, there has been a significant effort to utilize chemical and biochemical tools to influence intracellular pathways and thereby manipulate the behavior of cells and organisms.^[^ ^40^, ^41^ ^]^ However, once the cargos (small molecules, proteins, nucleic acids, bioconjugates, etc.) penetrate the cell, all control over it is relinquished.^[^ ^42^ ^]^ For example, it is challenging to manipulate the intracellular spatial distribution of the molecule, as well as the precise timing and endurance of its activity inside living organisms. On the other hand, the light‐activatable versions of these cargos has provided a unique and efficient way to regain control of the molecule, even after it has entered the cell. The noninvasive characteristic and remote activation through light irradiation of specific wavelengths, along with its simple control and quick, cost‐efficient operation, make this technique highly attractive. It allows the biological function of small‐molecule effectors, biomacromolecules, or drugs to be concealed, rendering them inactive until the photolabile moieties are removed upon light‐induced irradiation with a suitable wavelength.^[^ ^43^, ^44^, ^45^, ^46^, ^47^, ^48^ ^]^ We have previously developed the first photo‐activatable small‐molecular modulators toward the methyltransferase and demethylase of *N* ^6^‐methyladenine m^6^A RNA, respectively.^[^ ^49^, ^50^ ^]^ The biological activities of these compounds could be entirely concealed by attaching a photo‐removable moiety, which then rapidly cleaved upon a brief exposure to UV light. This method has facilitated a precise control over the significant hypermethylation or reduction of m^6^A RNA modification in transcriptome RNAs within live cells. However, the modulation of another catalog of biologically important epigenetic modification, i.e., histone methylation, in living cells through photo‐induced manipulation has not been realized yet. Building on our prior work in creating photo‐assisted bioorthogonal transformations for precise structural and functional control of RNA,^[^ ^51^, ^52^, ^53^, ^54^, ^55^, ^56^ ^]^ we aim to devise a new approach to regulate the histone modification process and consequently influence epigenetic marks in a spatiotemporal manner. Given that the activity of associated KMTs and KDMs differs across cell types and tissues, with specific enzymes being essential for various developmental processes, precise regulation of these enzymes would be extremely beneficial for preserving epigenome homeostasis and understanding their unique roles in gene expression regulation. And yet, the development of temporal and spatial methods for such regulation has not been achieved. We here present our recent advancement in the manipulation of the histone methylation in vitro and in living cells using a photocaged small organic molecule. This photo‐activatable chemical system allows an unprecedented and temporal inhibition of the nuclear KMT G9a, thus enabling a precise modulation of H3K9Me2 methylation in living cells without requiring any genetic manipulations (**Scheme** **1**A).

## 2 Results and Discussion

### 2.1 Design of Photocaged G9a Inhibitor by Molecular Docking

We chose G9a, the second histone methyltransferase discovered in mammals, as an example to investigate the potential of chemical modulation of HMTs. G9a oversees the mono‐ and di‐methylation of histone H3 lysine 9 (H3K9Me and H3K9Me2). It controls gene transcription by changing the strength of histone‐DNA binding through interaction with transcription factors.^[^ ^57^, ^58^ ^]^ Its increased levels have been observed in various cancer types and linked to negative prognosis.^[^ ^59^, ^60^ ^]^ Due to its distinct features, G9a has emerged as a highly promising target for anti‐cancer treatments. In the past decade, numerous G9a inhibitors have been identified as potential therapeutic agents for cancer.^[^ ^61^, ^62^ ^]^ However, there has not been a successful implementation of spatiotemporal modulation of G9a's activity. We first want to understand the influence of the chemical elements of small organic molecules on their biological activity, which would guide further optimization. For this purpose, we chose UNC0638 **1** (Scheme 1B), an inhibitor of G9a with excellent potency and selectivity but with poor pharmacokinetic (PK) properties,^[^ ^63^, ^64^, ^65^, ^66^, ^67^, ^68^ ^]^ and evaluated it in silico using docking and molecular dynamics simulations (Scheme 1C). Our computational investigations were consistent with the X‐ray cocrystal structure of the G9a‐**1**‐SAH complex^[^ ^63^ ^]^ that **1** did occupy the peptide binding groove but not interact with the SAM binding pocket (SAM: *S*‐adenosylmethionine). Indeed, the 1‐isopropylpiperidin‐4‐amino side chain of **1** was wrapped in the cavity composed of two Asp residues, in which the secondary *N*‐H of the quinazoline interacted with Asp1083 to form a key hydrogen bond that defined the potency and activity. Hence, we hypothesized and designed the photocaged G9a inhibitor **2** by attaching a (2‐nitrophenyl)ethyl carbamate group, a cascade photo‐caging motif, to the free *N*‐H (Scheme 1B). To facilitate the photo‐caged inhibitor design, we re‐analyzed the binding mode of inhibitor **2** with G9a in modeling studies, with which the computational profile of the newly designed inhibitor was compared with its precursor (Scheme 1C,D). As shown in Scheme 1C, in addition to the similar hydrogen bond formed between the *N* ^1^ of the quinazoline and the carboxylic acid of residue Asp1088, the piperidinyl moiety established a new interaction with the Asp1078. However, two crucial interactions for the G9a inhibitory activity control, the ^+^N‐H∙∙∙O between the pyrrolidinyl side chain with Leu1086 of the lysine binding channel and the aforementioned *N*‐H∙∙∙O(Asp1083), were totally abolished, as the photo‐caging (2‐nitrophenyl)ethyl carbamate group occupied the binding site and significantly distorted the quinazoline ring orientation and thus completely decreased the G9a recognition. In fact, the computational docking scores showed that the photo‐caged compound **2** could not bind to G9a protein where its precursor **1** occupied, exhibiting a much lower binding energy, inhibition constant, and ligand efficiency (Scheme 1D). These results suggest that blocking essential interactions between **1** and G9a using the photo‐caging group significantly reduces its ability to inhibit G9a.

### 2.2 Photophysical Properties of Photocaged G9a Inhibitor

**Scheme 2:**
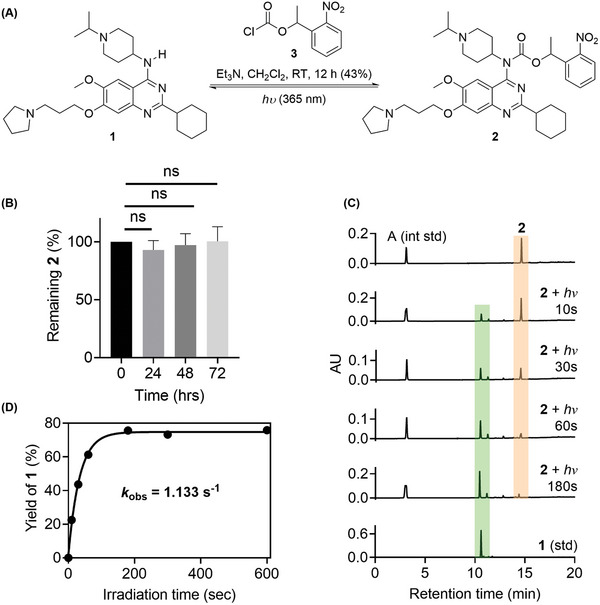
A) Chemical synthesis of photo‐caged inhibitor **2** and its photo‐release to **1** under light irradiation. B) Stability of compound **2** under dark in cell culture medium at 37 °C (*n* = 2). The error bars indicate the standard error of the mean (SEM), and pairs marked with ns = Not significant, analyzed by unpaired *t* test. C) HPLC profile for the de‐caging of **2** under light irradiation at different times. The authentical sample of **1** was also measured as reference. D) Measure of the photo‐release kinetics of **2**. The initial rate *k*_0_ was determined at 0–60 s times.

The photophysical properties of compound **2** were then studied (**Scheme** **2**). First, the caged inhibitor **2** was prepared by compound **1** reacting with 1‐(2‐nitrophenyl)‐1‐ethoxycarbonyl chloride **3** in an isolated yield of 43% (Scheme 2A), which was characterized by ^1^H/^13^C NMR and HRMS (see Supporting Information). Next, the stability under biological conditions and photo‐release kinetics of **2** was measured by high performance liquid chromatography (HPLC). To our delight, the (2‐nitrophenyl)ethyl carbamate‐caged inhibitor **2** exhibited superb stability in the dark and negligible degradation (Scheme 2B). Then the uncaging process of **2** was monitored in a 0.16 mm solution of methanol/water upon UV light irradiation at 365 nm (Scheme 2C). Adenosine (A) was used as the internal standard. Upon the 365 nm UV light irradiation, the anticipated product **1** occurred at the earliest time point measured (10 s). Over the time course (0–600 s), the intensity of the compound **1** gradually increased and reached the maximum at 180 s (HPLC yield 76%), indicating a completion of the uncaging processes (Scheme 2C,D). The initial rate of de‐caging of **2** was determined to be 1.133 s^−1^, which was fast enough to reduce the photo‐induced toxicity upon photolysis.^[^ ^45^, ^46^, ^48^ ^]^

### 2.3 Photo‐Regulation of G9a Activity In Vitro

**Scheme 3:**
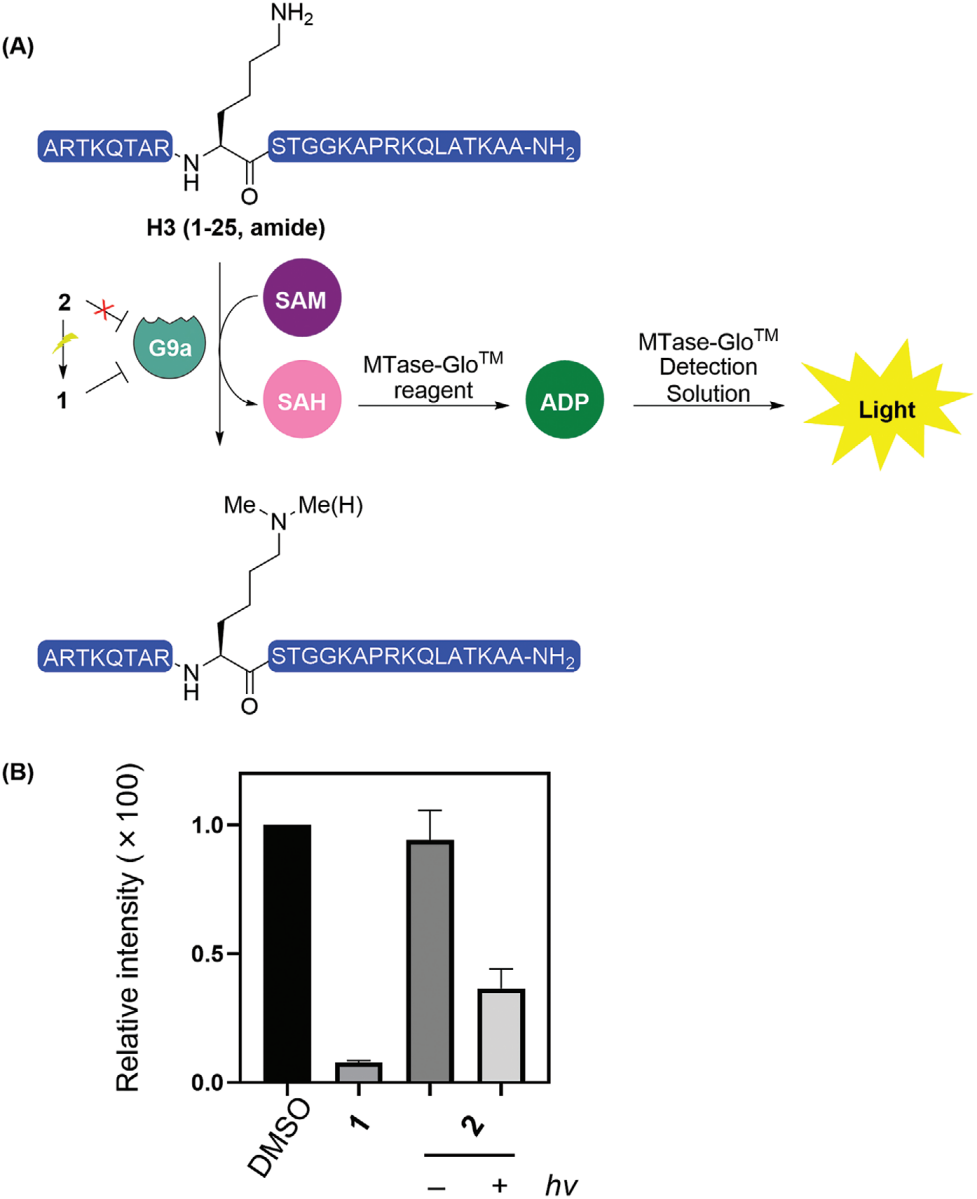
A) Inhibition of G9a under the photo‐induced activation of inhibitor **2** and the bioluminescence‐based assay to determine the inhibitory efficiency. B) Effects of light‐responsive activation of caged inhibitor **2** on G9a activity comparing with control groups of DMSO and compound **1** (*n* = 3). The error bars indicate the standard error of the mean (SEM).

To identify whether this photo‐stimuli‐responsive inhibitor **2** is applicable for modulation the methyltransferase G9a, the MTase‐Glo assay was used as a screening assay (**Scheme** **3**). The MTase‐Glo reagent measured the G9a activity through the transformation of the SAM‐dependent methylation reaction byproduct *S*‐adenosylhomocysteine (SAH) to adenosine triphosphate (ATP), which catalyzes the luciferase reaction to produce bioluminescent signal. Histone H3 (1‐25aa, amide), an *N*‐terminal peptide fragment of histone H3, was served as a substrate to validate the efficiency of photo‐activation by mimicking the H3K9 methylation (Scheme 3A).^[^ ^69^ ^]^ When monitoring the potency of inhibitor **1**, a 8% of bioluminescent signal was observed (Scheme 3B), which was consistent with previous in vitro studies that the G9a activity was completed inhibited by addition of **1**.^[^ ^63^ ^]^ Conversely, the (2‐nitrophenyl)ethyl carbamate substituted **2** showed a strong bioluminescent signal over the peptide tested (94% of the DMSO group), demonstrating that a photo‐caging at the *N*‐H site significantly abolished its methyltransferase inhibitory activity as expected (Scheme 3B). Nonetheless, a substantial recovery of the G9a inhibition was observed in the presence of light activation (37% of the DMSO group, 20 mW∙cm^−2^, 5 min, Scheme 3B). Taken together, these data demonstrated that conjugation of a photo‐liable group onto the HMT ligand is advantageous for temporarily masking its activity but could be re‐activated as needed upon a short time of light irradiation.

### 2.4 Photo‐Regulation of G9a Activity in Living Cells

**Scheme 4:**
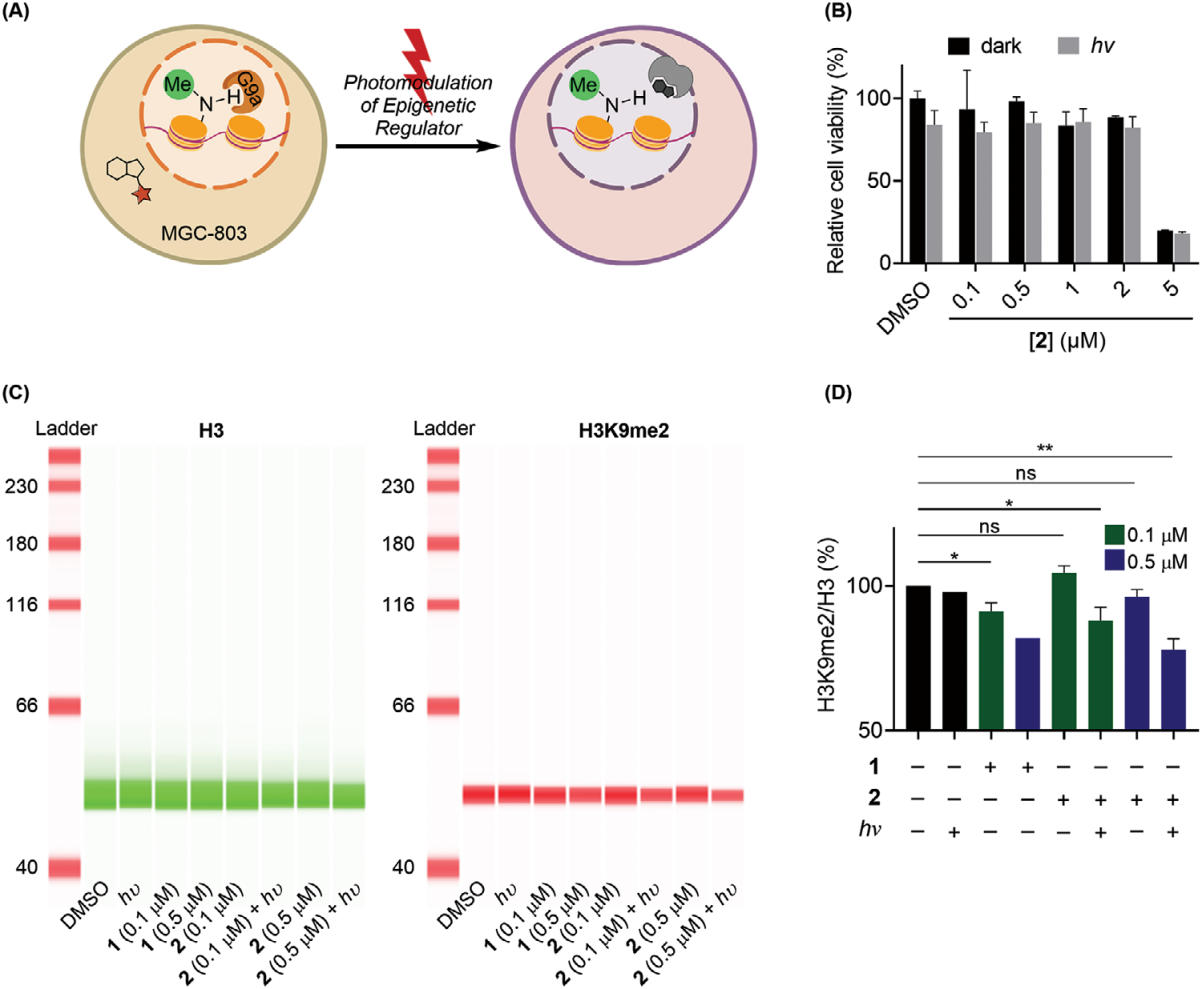
A) Schematics of photo‐responsive effects of G9a inhibition with compound **2** upon light irradiation toward the dimethylation of histone lysine in live MGC‐803 cell lines. B) Cell viability of caged inhibitor **2** with and without light irradiation. C) Representative digital western blot images of H3 protein and H3K9me2 levels. D) Quantification of three biological blot replicates of H3K9me2 levels (*n* = 3, except for *hυ* and 0.5 µm of **1** (*n* = 1)). The error bars indicate the standard error of the mean (SEM), and pairs marked with an * are significantly different at a *p*‐value ≤ 0.05, ** at *p*‐value ≤ 0.01. ns = not significant.

Inspired by the above in vitro results, we next addressed whether the photo‐caged inhibitor **2** could temporally modulate the G9a promoted methylation of epigenome histone lysine in living cells (**Scheme** **4**A). First, to validate the biocompatibility of the approach, we explored the release of the caged inhibitor **2** in the presence of living cells with and without light irradiation (*λ* = 365 nm, 20 mW∙cm^‒2^, 5 min). As shown in Scheme 4B, when the human gastric carcinoma cell line MGC‐803 was treated with caged inhibitor **2** (0.1–2 µm), no decrease in cell viability was observed. However, with the incubation of caged **2** (5 µm) for 72 h, a ca. 80% reduction in cell viability was detected. The loss of cell viability was similar to that observed when cells were directly treated with inhibitor **1** (2–5 µm) under the same conditions.^[^ ^64^ ^]^ There was no substantial influence on cell viability when cells were irradiated with light for 5 min in the presence of photo‐caged inhibitor **2** (0.1–2 µm). These results demonstrated that the photo‐caged HMT inhibitor can be utilized for the light‐triggered in vivo release of bioactive compounds.

Next, the intracellular photo‐activation of caged **2** and its inhibitory effect toward the G9a induced methylation of H3K9 was investigated (Scheme 4C). Changes in the dimethylation of H3K9 (H3K9me2) were assessed using the digital western blotting imaging with infrared (IR) and near‐infrared (NIR) fluorescence.^[^ ^70^, ^71^ ^]^ In MGC‐803 cells treated with inhibitor **1**, a slight reduction of H3K9me2 could be detected (9%, 0.1 µm), which was significantly amplified (ca. twofold, 18%) by further treatment with 0.5 µm of **1** (Scheme 4D). When similar cells were treated with compound **2** (0.1 and 0.5 µm), a fluorescence signal of H3K9me2 comparable to the control group was observed, indicating that the photo‐caged molecule by itself did not disrupt the catalytic activity of G9a. However, upon irradiation of the cells (*λ* = 365 nm, 20 mW∙cm^‒2^, 5 min), a 16% and 18% decrease in H3K9me2 fluorescence was observed at 0.1 and 0.5 µm, respectively, which was similar to that observed when cells were directly treated with inhibitor **1** (0.5 µm). Importantly, living cells exposed to similar irradiation conditions did not show any change in H3K9me2 level, ruling out the direct light effect on the dimethylation process. Taken together, these results clearly substantiated our notion that the caged inhibitor was not active in living cells, until it was activated by irradiation, and the activated inhibitory effects were comparable to the direct treatment with its precursor.

## 3 Conclusion

Histone methylation has a critical and well‐conserved role in embryo development by regulating the expression of specific genes. The location of the gene determines its spatiotemporal expression patterns: genes at the far ends of the clusters are usually expressed later in development and are limited to the caudal regions of the embryo.^[^ ^3^ ^]^ Besides, previous research in mice have revealed that the activity of KMTs and KDMs varies between cell types and tissues. For instance, the methyltransferase EZH1/2 is crucial for neural and heart development, while MLL4, LSD1, and G9a, a representative methyltransferase that could be selectively modulated here, regulate muscle generation, fat generation, and hematopoiesis. On the other hand, many KMTs are highly expressed in cancer or other diseases, and precise regulation of those proteins can improve the efficiency while reducing the toxic side effects on normal cells and tissues. Therefore, a precise temporal and spatial regulation of KMTs is essential for epigenome homeostasis. Consequently, the development of chemical and biochemical tools capable of specifically regulating epigenome modifying enzymes at specific times, cells, or tissues can aid in understanding the series of life processes in which the enzymes are involved. However, it remains a significant challenge to develop precise temporal and spatial approaches for such regulation.

Here we have demonstrated a new strategy for controlling KMTs’ activity with light using a photo‐caged small organic molecule that enabled temporal modification of live‐cell histone methylation without any genetic manipulations. G9a is an essential epigenetic protein that plays a crucial role in controlling gene expression by altering chromatin structure. Our research has shown that a photo‐caged inhibitor **2** can effectively regulate its methyl transfer activity through a rapid photo‐release process. Given the stability of (2‐nitrophenyl)ethyl carbamate‐caged molecule even in living cellular conditions, we expect it will find broad application as a general photo‐liable group for epigenome manipulation, and this technique should be readily and widely applicable at a variety of nucleus locations to address a range of biological questions.

## Conflict of Interest

The authors declare no conflict of interest.

## Author Contributions

This project was conceived by C.S.W. and L.C.; C.S.W. and L.C. designed the experiments; C.S.W. and X.S. performed experiments; X.S. performed docking analysis; C.S.W. performed the statistical analysis; C.S.W. and L.C. wrote the manuscript; L.L. provided ongoing mentoring to refine the studies; C.S.W. and L.C. edited the manuscript. All authors agreed with the final version of this manuscript.

## Supporting information

Supporting Information

## Data Availability

The data that support the findings of this study are available in the supplementary material of this article.
