# Supplementary material for: A Live‐Cell Epigenome Manipulation by Photo‐Stimuli‐Responsive Histone Methyltransferase Inhibitor
Source: Adv Sci (Weinh). 2024 Sep 9;11(41):2404608. doi: 10.1002/advs.202404608 (PMC11538670; doi:10.1002/advs.202404608)

Supporting Information

A Live-Cell Epigenome Manipulation by Photo-stimuli-responsive Histone Methyltransferase Inhibitor

*Chuan-Shuo Wu, Xin Sun, Li Liu and Liang Cheng**

1. General information

Unless otherwise noted, all reagents were obtained from commercial suppliers and were used without further purification. The ^1^H NMR and ^13^C NMR were collected by Bruker 300 or 500 MHz spectrophotometer. Chemical shifts δ are given in ppm relative to the residual proton signals of the deuterated solvent for ^1^H and ^13^C NMR (CDCl_3_, ^1^H NMR: 7.26 ppm; ^13^C NMR: 77.0 ppm). Multiplicities are reported as follows: singlet (s), doublet (d), doublet of doublets (dd), triplet (t), quartet (q), multiplet (m). HRMS were obtained using electrospray ionization (ESI) mass spectrometer (Thermo Fisher Scientific, Exactive). HPLC data was collected with Waters ACQUITY H-Class equipped with a Sunfire^®^ C18 5 μm (4.6 × 250 mm) column.

2. Procedures for the synthesis of compound 2

**Figure S1**. the synthesis of compound **2**

To a solution of 1-(2-nitrophenyl)ethanol (**S1**, 500 mg, 3 mmol) in THF (10 mL) were added Na_2_CO_3_ (381.6 mg, 3.6 mmol) and triphosgene (1.78 g, 6 mmol) under argon atmosphere. After stirring 24 h at room temperature, H_2_O (20 mL) was added at 0 °C, and the mixture was extracted with CH_2_Cl_2_ (3 × 20 mL). Combined organic layer was dried over Na_2_SO_4_ and concentrated under reduced pressure, to give **S2** as a colorless oil which was used for the next reaction without purification. ^1^H NMR (300 MHz, CDCl_3_) δ 8.03 (d, *J*= 8.1 Hz, 1H), 7.79–7.65 (m, 2H), 7.57–7.46 (m, 1H), 6.46 (q, *J*= 6.4 Hz, 1H), 1.77 (d, *J*= 6.5 Hz, 3H).

To a solution of UNC0638 (51 mg, 0.1 mmol) in DCM (0.8 mL) were added **S2** (69 mg, 0.3 mmol) and Et_3_N (30.3 mg, 0.3 mmol). After stirring 12 hours at room temperature, saturated Na_2_CO_3_ aqueous solution (5 mL) was added and the mixture was extracted with CH_2_Cl_2_ (3 × 5 mL). Combined organic layer was dried over Na_2_SO_4_ and concentrated under reduced pressure, then purified via silica gel column chromatography to give **2** as pale yellow foamy solid (30.2 mg, 43%). ^1^H NMR (500 MHz, CDCl_3_) δ 7.90 (t, *J* = 9.5 Hz, 1H), 7.67–7.55 (m, 2H), 7.45–7.35 (m, 1H), 7.21–7.07 (m, 1H), 6.85 (s, 1H), 6.29–6.18 (m, 1H), 5.26 (d, *J* = 7.0 Hz, 1H), 4.33–4.22 (m, 1H), 4.22–4.02 (m, 2H), 3.95 (s, 3H), 3.64–3.44 (m, 2H), 3.35–3.24 (m, 2H), 3.01 (d, *J* = 11.6 Hz, 2H), 2.95–2.83 (m, 1H), 2.78–2.68 (m, 1H), 2.65 (s, 2H), 2.47 (t, *J* = 10.7 Hz, 2H), 2.25 (d, *J* = 11.1 Hz, 2H), 2.20–2.09 (m, 2H), 1.99 (d, *J* = 12.1 Hz, 2H), 1.85 (d, *J* = 12.4 Hz, 2H), 1.90–1.52 (m, 14H), 1.49–1.34 (m, 3H), 1.14 (d, *J* = 6.6 Hz, 6H). ^13^C NMR (125 MHz, CDCl_3_) δ 169.4, 157.8, 155.0, 153.2, 148.3, 147.1, 138.6, 133.5, 128.1, 128.0, 127.0, 124.3, 108.3, 106.8, 99.9, 69.3, 69.2, 66.0, 65.9, 56.4, 56.3, 54.9, 48.1, 47.8, 47.8, 46.7, 46.7, 43.85, 32.1, 31.9, 26.4, 26.2, 22.2, 18.3. HRMS calculated for C_39_H_54_N_6_O_6_ [M+HCl+H]^+^: 739.3944, found: 739.3942.

3. Molecular docking

AutoDock 4.2 (The Scripps Research Institute, Molecular Graphics Laboratory) was used to find out binding sites and binding energies of ligands (UNC0638 and **2**) with the receptor (G9a). Firstly, we downloaded the crystal structure of G9a with the *S*-adenosyl-*l*-homocysteine (SAH) and UNC0638 (PDB: 3RJW) from Protein Data Bank, then extracted G9a as the receptor by PyMOL (The PyMOL Molecular Graphics System, Version 2.3.2, Schrödinger, LLC) molecular graphics software. The kinetic energy optimization of ligands were carried out by the MM2 function in Chem 3D to obtain their PDB files. The solvent water molecules of receptor were deleted and polar hydrogen were added. The docking mode is semi-flexible docking. The binding site was surrounded with a grid-box sized 38 × 54 × 28 (X center: 18.903, Y center: 11.488, Z center: 7.117) points with a grid spacing of 0.375 Å. The AutoDock4.2 force field was used in all molecular docking simulations and the Lamarckian Genetic Algorithm (LGA) was chose. All calculations were performed on an ADOL14UA5700-0DAUXAJX10 based machine running Windows 11 as the operating system. The model and the diagram were generated with PyMOL molecular graphics software.

|  | UNC0638 | **2** |
| --- | --- | --- |
| Binding energy | -11.05 | -9.38 |
| Ligand efficiency | -0.3 | -0.18 |
| Inhibit constant | 8.01 nm | 133.92 nm |
| Intermol energy | -14.03 | -13.25 |
| Desolv energy | -11.51 | -12.12 |
| Total internal | -1.3 | -5.08 |
| Torsional energy | 2.98 | 3.88 |
| Unbound energy | -1.3 | -5.08 |

**Table S1**: Molecular docking of G9a with UNC0638 and **2**

4. Stability of 2

**Figure S2**. Stability of **2** under cell culture at 37°C with different incubation time

A mixed solution of **2** (1 mm, 50 μL) and high-glucose dulbecco's modified eagle medium (DMEM, Gibco, 50 μL), supplemented with 10% fetal bovine serum (FBS, Gibco) and 1% penicillin/streptomycin (Gibco), were incubated in 37°C with different time (0 h, 24 h, 48 h, 72 h), respectively. After incubation, 1 mL cold acetone was added to the solution and centrifugated at 15000 g for 5 minutes. The supernatant was concentrated by vacuum. *N*^6,6^-dimethyladenosine (m^6,6^A, 1 mm, 50 μL) was used as the internal standard and added to the supernatant. The solution was diluted with CH_3_OH (150 μL) and then subjected to HPLC analysis. The area ratio of **2** with m^6,6^A was almost the same after 24 h, 48 h, 72 h incubation. UNC0638 was not observed. HPLC conditions: The detection wavelength was set as 254 nm. 0.1% trifluoroacetic acid in H_2_O (buffer A) and 0.1% trifluoroacetic acid in CH_3_OH (buffer B) were applied as mobile phase. A gradient of 5% B (0-2 min), 5%-100% B (2-15 min), 100% B (15-22 min), 100%-5% B (22-25 min), 5% B (25-27 min) was used. Flow rate: 1 mL/min.

5. Monitor photo-release of 2 by HPLC

5.1 Determination of internal standard curve

Adenosine was used as the internal standard. A mixed solution of UNC0638 and adenosine with different concentration ratio (0.1, 0.2, 0.4, 0.6, 0.8, 1) was prepared and then subjected to HPLC analysis. According to the peak area ratio of each solution, the following internal standard curves were obtained. HPLC conditions: The detection wavelength was set as 254 nm. 0.1% trifluoroacetic acid in H_2_O (buffer A) and 0.1% trifluoroacetic acid in CH_3_OH (buffer B) were applied as mobile phase. A gradient of 20% B (0-2 min), 20%-100% B (2-15 min), 100% B (15-22 min), 100%-20% B (22-25 min), 20% B (25-27 min) was used. Flow rate: 1 mL/min.

**Figure S3**. internal standard curve of UNC0638 and **A**

5.2 Photo-release of 2

To a solution of **2** in methanol (1 mm, 1 mL) were added adenosine in methanol (1 mm, 1 mL), methanol (1 mL), H_2_O (3 mL), the solution was fully mixed well by oscillation. Taking 200 μL of the solution and divided it into different 1.5 mL centrifuge tubes. Irradiating those centrifuge tubes under 365 nm light for different time (0 s, 10 s, 30 s, 60 s, 180 s, 300 s, 600 s) and then subjected to HPLC analysis. HPLC condition was same as above. According to the peak area ratio of each solution and the internal standard curve, the yield of UNC0638 can be obtained. The generation of UNC0638 was confirmed through pre-HPLC and mass spectrometry.

**Figure S4**. mass spectrometry of 10.4 min after pre-HPLC. HRMS calculated for UNC0638 [M+H]^+^: 510.3803, found: 510.3817. [M+HCl+H]^+^: 546.3569, found: 546.3585

6. Expression of G9a (913-1193aa)-6x His ^[1]^

DNA fragment encoding the methyltransferase domain of human G9a (913-1193aa) was synthesized and cloned into the pet24a vector, downstream of the poly-histidine coding region. The sequencing was correct. Transform the plasmid into BL21(DE3) and induce its expression by addition of 400 μm isopropyl-1-thio-D-galactopyranoside for overnight at 22 °C. The protein was purified as following: harvested cells were resuspended in Ni-IDA Binding buffer (20 mm Tris-HCl, 20 mm imidazole, 0.5 m NaCl, pH 8.0) supplemented with Triton-100 and phenylmethyl sulfonyl fluoride. The cells were lysed by intermittent sonication. The supernatant was loaded onto Biologic LP system equipped with Ni-IDA-Sepharose CL-6 column. The column was sequentially washed with 20 mm Tris-HCl (pH 8.0), containing 250 mm NaCl and different concentrations of imidazole (50, 100, 400, 600 mm). Those effluents were characterized by 12% SDS-PAGE. The effluent corresponding to lane 5 was collected, then purified with g25 molecular sieve and replaced the buffer with 20 mm Tris-HCl (pH 8.0), containing 150 mm NaCl and 10% glycerol. The final concentration was 0.4 mg/mL (3 mL). Storing it at ‒80 °C until use.


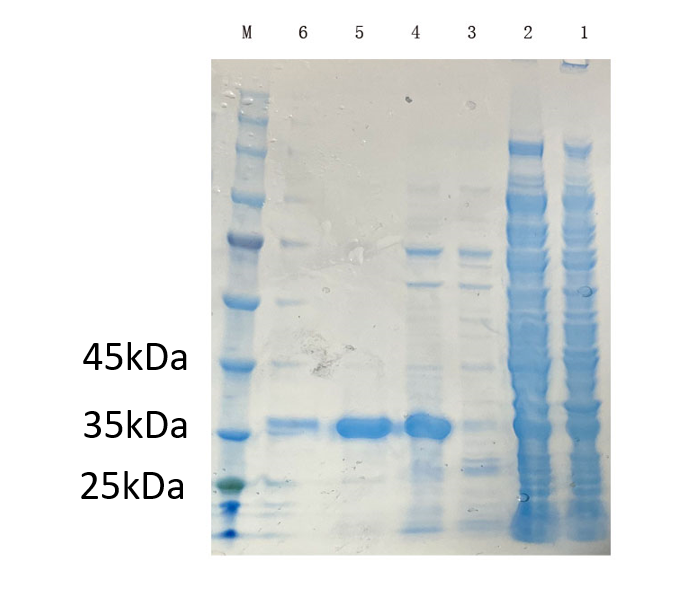


**Figure S5**. Expression of G9a (913-1193 aa)-6x His. M: Ladder; Lane 1: Whole cell; Lane 2: broken supernatant; Lane 3-6: Ni NTA affinity purification with 50 mm imidazole, 100 mm imidazole, 400 mm imidazole, 600 mm imidazole elution.

7. Histone Methyltransferase Assay

The MTase-Glo^TM^ Methyltransferase Assay Kit (Promega, V7601) was employed to assay the activity of G9a treated with UNC0638 and **2** under light or dark. After the methyltransferase reaction was complete, the MTase-Glo™ Reagent could convert SAH to ADP. The MTase-Glo™ Detection Solution was then added to convert ADP to ATP, which was detected *via* a luciferase reaction.^[2]^

The experiments were conducted in 1X reaction buffer (20 mm Tris-HCl pH = 8.0, 50 mm NaCl, 1 mm EDTA, 1 mm DTT, 0.1mg/mL BSA, 3 mm MgCl_2_). To a solution of G9a (25 nm, 5 μL diluted with 1X reaction buffer and MTase-Glo™ Reagent 10x) was added 1X reaction buffer as control or 5 μm UNC0638, **2** (5 μL, DMSO content, constituted 1%). After 10 min incubation (the solution which needs *hv*, was subjected to 5 minutes of UV illumination at λ = 365 nm (20 mW∙cm^‒2^) then incubated for additional 5 minutes), 50 μm H3 (1-25 aa, amide) (purchased from Sangon, 5 μL) and 62.5 μm of *S*-(5′-adenosyl)-*l*-methionine (SAM, 10 μL) provided by MTase assay kit was added to initiated the enzymatic reaction. After 60min incubation at room temperature, MTase-Glo™ detection solution (25 μL) was added and then reacted 60 min. Finally, measure luminescence using a plate-reading luminometer (Bio Tek Synergy H1).

8. Cell culture and CCK-8 assay

MGC-803 cells were maintained in high-glucose dulbecco's modified eagle medium (DMEM, Gibco) supplemented with 10% fetal bovine serum (FBS, Gibco) and 1% penicillin/streptomycin (Gibco) at 37 °C in the presence of 5% CO_2_. CCK-8 cell proliferation and cytotoxicity assay kit (CA1210) was purchased from Beijing Solarbio Science & Technology co., Ltd. Follow the instruction of kit, MGC-803 cells were seeded at a density of 5 × 10^3^ cells per well (100 μL) into two 96-well plates. After 24 h plating, cells were treated in fresh medium with the photocaged molecules at varying concentrations (0, 100 nm, 500 nm, 1 μm, 2 μm, 5 μm. 100 μL, 1% DMSO). One plate was incubated for 48 hours at 37 °C. While another plate was subjected to 5 minutes of UV illumination at λ = 365 nm (20 mW∙cm^‒2^) at 25 °C, then incubated for additional 48 hours at 37 °C. After 48 h plating, cells were washed twice with PBS and added 100 μL fresh medium containing 10% CCK-8 solution. Incubate those plates for 1 hours in the incubator. Measure the absorbance at 450 nm using a microplate reader (Bio Tek Synergy H1).

9. Cell experiment and Histone extraction

MGC-803 cell culture was the same as above. MGC-803 cells were seeded at a density of 5 × 10^6^ cells into 10 cm cell culture dishes. After 24 h incubating, cells were treated in fresh medium with UNC0638 or the photocaged molecules at varying concentrations (0, 100 nm, 500 nm, 1% DMSO). Those dishes that do not need *hv* were incubated for 72 hours at 37 °C. While other dishes were subjected to 5 minutes of UV illumination at λ = 365 nm (20 mW∙cm^‒2^) at 25 °C after 24 h incubating, then incubated for additional 48 hours at 37 °C.

According to the lecture^[3]^, histones were extracted from MGC-803 cells. Cells were harvested at the appropriate time and lysed by 30 minutes incubation on ice in 1 mL nuclear extraction buffer (10 mm Tris‐HCl, 10 mm MgCl2, 25 mm KCl, 1% Triton X-100, 8.6% sucrose, protease inhibitor cocktail (Sangon, C600386)). Nuclei were collected by centrifugation at 1000 g for 5 minutes at 4 °C. Supernatant was removed and histones extracted for 4 hours with 0.4 N cold sulfuric acid (400 μL). Extracts were clarified by centrifugation at 15000 g for 5 minutes at 4 °C and transferred to fresh microcentrifuge tube. 133 μL 100% TCA solution was added to each tube. Histones were precipitated at 4 °C overnight, pelleted by centrifugation at 15000 g for 5 minutes, washed twice with cold acetone and resuspended in 100 μL water. Histones were quantified using the BCA protein assay (Sangon, C503051).

10. Detection of H3K9me2/H3 by Simple Western

The method was adopted according to the protocol provided by the manufacturer (Protein simple, Jess). To determine suitable sample detection concentration and antibody dilution, a pre-experiment was conducted using a sample with known protein concentration, and standard curves were established between H3K9me2 and NIR luminescence signal area, as well as between H3 and IR luminescence signal area. We found that there is a good linear relationship between the NIR signal corresponding to H3K9me2 at an antibody dilution of 1:10 and a total protein concentration of 0.1-0.9 mg/mL; The IR signal corresponding to H3 has a good linear relationship between antibody dilution of 1:10 and total protein concentration of 0.033-0.3 mg/mL. So we determined a suitable sample detection concentration between 0.2-0.3 mg/mL, with an antibody dilution of 1:10.

**Figure S6**. standard curve of IR and NIR with total protein concentration

After determined suitable sample detection concentration and antibody dilution, we compared the levels of H3K9me2 under different treatment methods, using H3 as loading control. The exacted histone solutions were diluted with 1X sample buffer and 5X master mix to achieve a protein concentration of 0.24 mg/mL. In total, 12-230 kDa fluorescence separation module (SM-FL001) was used. H3K9me2 was detected by mouse anti-histone H3 antibody (Abcam, 1220) while H3 was detected by rabbit anti-histone H3 antibody (Abcam, 1791) with an antibody dilution of 1:10, respectively. Anti-mouse NIR detection module and anti-rabbit IR detection module were used according to manufacturer's instructions. Compass software (Protein Simple) was used to visualize virtual gels. Relative protein quantification was generated from chromatograms of the indicated samples and corrected through standard curve.

11. Statistical analysis

Statistical analysis was performed using Prism 8.4.2 (GraphPad Software). Student’s *t*-test was used for comparisons between two groups. For comparisons among multiple groups, one-way ANOVA was used. Unless other noted, all the experiments were performed at least three times. **p* < 0.05, ***p* < 0.01, ****p* < 0.001, *****p* < 0.0001, n.s., not significant.

12. Reference

1. H. Wu, J. Min, V. V. Lunin, T. Antoshenko, L. Dombrovski, H. Zeng, A. Allali-Hassani, V. Campagna-Slater, M. Vedadi, C. H. Arrowsmith, A. N. Plotnikov, M. Schapira, *PLoS One* 2010, *5*, e8570.
2. K. Hsiao, H. Zegzouti, S. A. Goueli, *Epigenomics* 2016, *8*, 321.
3. S. R. Daigle, E. J. Olhava, C. A. Therkelsen, C. R. Majer, C. J. Sneeringer, J. Song, L. D. Johnston, M. P. Scott, J. J. Smith, Y. Xiao, L. Jin, K. W. Kuntz, R. Chesworth, M. P. Moyer, K. M. Bernt, J.-C. Tseng, A. L. Kung, S. A. Armstrong, R. A. Copeland, V. M. Richon, R. M. Pollock,*Cancer Cell* 2011, *20*, 53.

**13. NMR spectra**

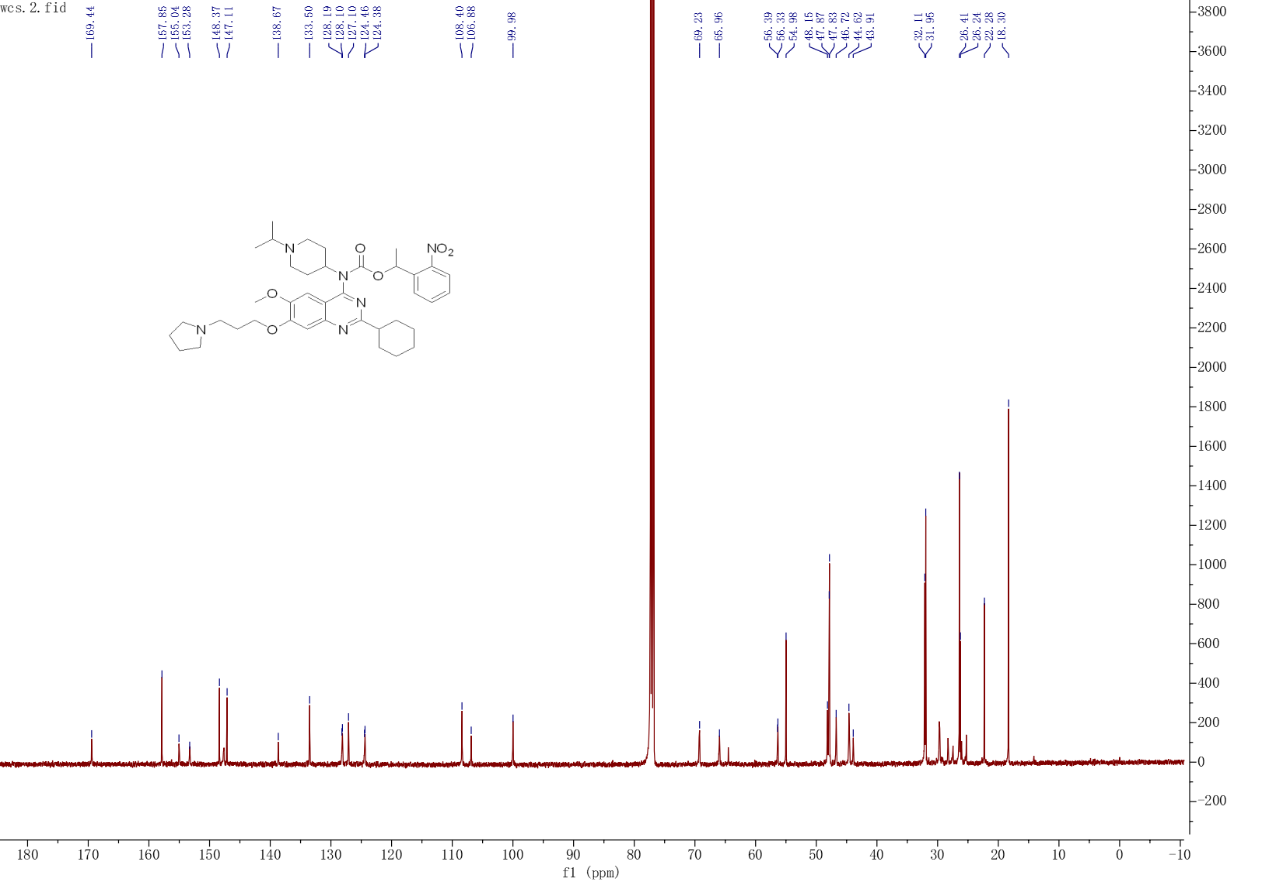

Supplement: Supplementary file 1 — Supporting Information [file ADVS-11-2404608-s001.docx]
